# Attenuation of PM2.5-Induced Lung Injury by 4-Phenylbutyric Acid: Maintenance of [Ca^2+^]i Stability between Endoplasmic Reticulum and Mitochondria

**DOI:** 10.3390/biom14091135

**Published:** 2024-09-08

**Authors:** Zhenhua Ma, Xiaohui Du, Yize Sun, Yunna Jia, Xiaojun Liang, Yunhang Gao

**Affiliations:** 1 College of Animal Science and Technology, Jilin Agricultural University, Changchun 130118, China; mazhenhua1030@163.com (Z.M.); duxiaohui2001@163.com (X.D.); sunyize1228@163.com (Y.S.); 15590074644@163.com (Y.J.); 2 Institute of Animal Science, Ningxia Academy of Agriculture and Forestry Sciences, Yinchuan 750002, China

**Keywords:** PM2.5, 4-PBA, intracellular Ca^2+^, ER stress, mitochondrial damage, pyroptosis

## Abstract

Fine particulate matter (PM2.5) is a significant cause of respiratory diseases and associated cellular damage. The mechanisms behind this damage have not been fully explained. This study investigated two types of cellular damage (inflammation and pyroptosis) induced by PM2.5, focusing on their relationship with two organelles (the endoplasmic reticulum and mitochondria). Animal models have demonstrated that PM2.5 induces excessive endoplasmic reticulum stress (ER stress), which is a significant cause of lung damage in rats. This was confirmed by pretreatment with an ER stress inhibitor (4-Phenylbutyric acid, 4-PBA). We found that, in vitro, the intracellular Ca^2+^ ([Ca^2+^]i) dysregulation induced by PM2.5 in rat alveolar macrophages was associated with ER stress. Changes in mitochondria-associated membranes (MAMs) result in abnormal mitochondrial function. This further induced the massive expression of NLRP3 and GSDMD-N, which was detrimental to cell survival. In conclusion, our findings provide valuable insights into the relationship between [Ca^2+^]i dysregulation, mitochondrial damage, inflammation and pyroptosis under PM2.5-induced ER stress conditions. Their interactions ultimately have an impact on respiratory health.

## 1. Introduction

Fine particulate matter (PM2.5) is a significant air pollutant. Long-term exposure to PM2.5 poses a considerable risk to human health due to its small specific surface area and high adsorption rate for harmful substances [1]. Many respiratory diseases such as allergic airway diseases, asthma and chronic obstructive pulmonary disease (COPD) have been associated with exposure to PM2.5 [2]. Therefore, studying the interaction between PM2.5 and the respiratory barrier is crucial in deciphering the toxicity of PM2.5, particularly for the respiratory system. At the same time, the interaction between the respiratory barrier and particulate matter makes the potential mechanism of alveolar macrophage damage a key factor in assessing the respiratory toxicity of PM2.5.

In certain specific settings, PM2.5 can be even more detrimental. In animal farming environments, for example, PM2.5 concentrations are higher, the composition is more complex, and the widespread presence of pathogenic microorganisms represents a greater threat to the respiratory health of animals and workers [3]. This was previously demonstrated in our studies on the characterization and composition of PM2.5 in cowsheds [4]. Previous research has demonstrated that PM2.5 levels in cowsheds can significantly disrupt intracellular Ca^2+^ ([Ca^2+^]i) homeostasis in alveolar macrophages. The cellular inflammatory response and apoptosis caused by PM2.5 can be attenuated by pretreatment with a [Ca^2+^]i antagonist (BAPTA-AM). The cytotoxicity induced by PM2.5 is associated with disorders in [Ca^2+^]i [5]. However, the cause of the disruption in [Ca^2+^]i during this process and the pathway by which cellular inflammation and death are accelerated remain unclear.

A close relationship exists between two crucial intracellular calcium reservoirs, the endoplasmic reticulum and the mitochondria, in the context of the biological function of Ca^2+^ as a second messenger [6]. The endoplasmic reticulum (ER) acts as a major calcium reservoir in the cell, storing large amounts of Ca^2+^. The ER can release Ca^2+^ into the cytoplasm when the cell is exposed to certain stimuli. Mitochondria are able to sense changes in Ca^2+^ concentration in the cytoplasm and take up Ca^2+^ via specific calcium transport proteins. The ER and mitochondria are situated in close proximity to one another within the cell, and their proximity allows for the formation of specific structural regions. This proximity enables functional communication between the two organelles. The interaction between the ER and mitochondria is altered when cells are stimulated by various stressors (such as oxidative stress, hypoxia, or toxins) [7]. Mitochondria-associated membranes (MAMs) play a crucial role in facilitating mitochondrial Ca^2+^ uptake and regulating the activities of key enzymes in the tricarboxylic acid cycle. Abnormal mitochondrial Ca^2+^ uptake mediated by MAMs has been reported to be associated with a variety of diseases, including metabolic diseases and neurodegenerative pathologies [8]. Calcium signaling is a fundamental process that regulates a multitude of physiological functions within the cell. Varying degrees of damage to subcellular structures including the endoplasmic reticulum, mitochondria, lysosomes and nucleus inevitably cause cell death.

Several studies have indicated that PM2.5 is capable of inducing endoplasmic reticulum stress (ER stress) in a wide range of in vitro and in vivo models [9,10]. The ER plays a pivotal role in the process of cellular protein synthesis and folding. In pathological conditions or in response to environmental stresses, the processes of protein synthesis and folding are disrupted, resulting in the accumulation of unfolded or misfolded proteins within the lumen of the ER. If the ER stress process described above persists and cannot be alleviated, this will lead to an abnormal physiological environment and rapid cell death [11]. Recent studies have indicated a significant correlation between pyroptosis, a form of programmed cell death, and PM2.5-induced cellular damage [12]. The initial stages of pyroptosis are characterized by cell swelling, pore formation in the cell membrane and subsequent cell rupture, accompanied by significant morphological alterations. These changes are often accompanied by a robust inflammatory response, which may result in tissue damage and the exacerbation of disease [13]. Nevertheless, the questions of whether endoplasmic reticulum stress is an important cause of PM2.5-induced lung injury and whether the relationship between ER stress and alveolar macrophage pyroptosis is related to [Ca^2+^]i disruption remain unanswered.

In this study, a model for rat whole-body PM2.5 exposure and an in vitro model for rat alveolar macrophage exposure were constructed. A known inhibitor of ER stress (4-Phenylbutyric acid, 4-PBA [14,15]) was used with the aim of exploring the role and potential relationship of ER stress in PM2.5-induced lung injury, as well as in two types of cellular damage (inflammation and pyroptosis).

## 2. Materials and Methods

### 2.1. Materials

In this study, 4-PBA (MCE, Monmouth Junction, NJ, USA) was dissolved in DMSO to obtain a final concentration of 1 mM. Antibodies against GAPDH, NLRP3 and GSDMD were purchased from ABclonal, Inc., Boston, MA, USA; antibodies against Caspase-1 were purchased from Abcam Limited, Britain; antibodies against cleaved Caspase-1 were purchased from Cell Signaling Technology, Inc., Boston, MA, USA; and antibodies against ATF6, CHOP, GRP78, Cyt-c, ASC, and secondary antibodies (Goat Anti-Rabbit IgG) were purchased from Proteintech Group, Inc., Chicago, IL, USA.

### 2.2. PM2.5 Sample Preparation

PM2.5 samples were collected from cattle farms using an intelligent TSP sampler (2030, Qingdao Lonying Environmental Technology Co., Ltd., Qingdao, Shandong, China). Specific details of the methods have been detailed in previous research, and previous studies have also characterized and analyzed PM2.5 samples for their chemical composition [4,5].

### 2.3. Animal Model Establishment and Grouping

**Figure 1:**
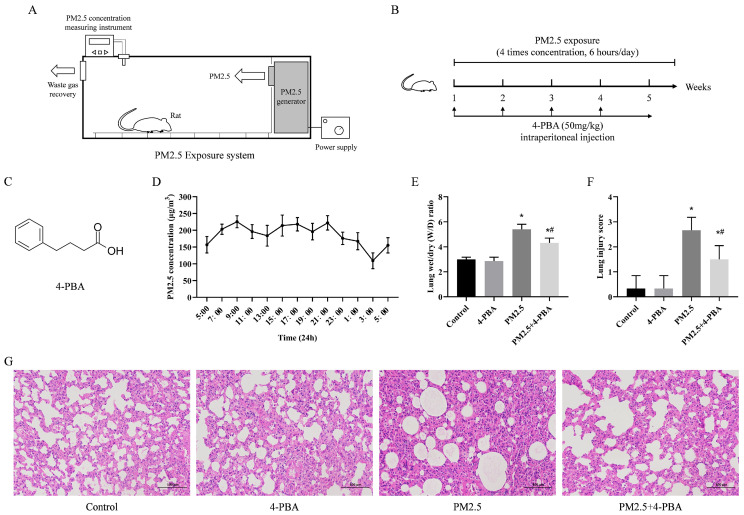
4-PBA inhibits PM2.5-induced lung injury in rats. (**A**) Schematic diagram of the rat whole-body PM2.5 exposure apparatus. (**B**) Schematic diagram of the in vivo experiment. (**C**) The chemical structural formula of 4-PBA. (**D**) Daily variation in PM2.5 concentration in cowsheds. (**E**) W/D ratio in rat lungs. (**F**) Pathology changes scored accordingly. (**G**) H&E sections of rat lungs after PM2.5 exposure and 4-PBA treatment. Scale bar = 100 μm. Results are expressed as the mean ± SD of triplicate determinations. * *p* < 0.05, compared with the Control group; ^#^ *p* < 0.05, compared with the PM2.5 group.

In the experiment, 6-week-old SPF-grade SD rats (Liaoning Changsheng Biotechnology Co., Ltd., Benxi, Liaoning, China) were used for PM2.5 respiratory exposure experiments. Following a one-week adaptation period under SPF conditions, the rats were placed in a whole-body PM2.5 exposure apparatus. The rat is an ideal model animal for studying respiratory diseases. The study team developed, optimized, and built a whole-body PM2.5 exposure apparatus for rats based on their body size. The apparatus is made up of three main components: a PM2.5 generator, an exposure chamber, and a module for measuring PM2.5 concentrations in real-time. The whole-body exposure apparatus can be used to expose rats to filtered clean air or concentrated PM2.5. The concentration of PM2.5 inside the exposed apparatus can be measured and adjusted in real-time. Therefore, animal modeling using this apparatus can reflect real human or animal exposure to ambient PM2.5, providing more accurate data. Rats were subjected to PM2.5 exposure using the procedure outlined in References [16,17]. Briefly, the PM2.5 concentration in the actual environment was measured, and the rats were exposed to 4 times the concentration for 6 h per day for 30 days. Based on the respiratory coefficient of an adult rat (200 g), the single breath volume (0.86 mL), and the respiratory rate (85 breaths/min) of adult rats, the daily exposure to PM2.5 was calculated to be approximately 78.246 μg. The exposure process is shown in Figure 1A,B.

During the experiment, the animals were allowed to drink and feed freely, and their body weight was not artificially controlled. The rats were divided into four different treatment groups. The group of rats exposed to clean air was designated as the Control group and did not receive any additional treatment. The group of rats that received only 4-PBA injections (50 mg/kg) was designated as the 4-PBA group. Rats exposed to four times the ambient atmospheric concentration of PM2.5 for 6 h per day were defined as the PM2.5 group. Rats receiving the same PM2.5 exposure after pre-injection with 4-PBA were defined as the PM2.5 + 4-PBA treatment group. After 30 consecutive days of exposure, blood samples were collected from the abdominal aorta of rats following the administration of 3% sodium pentobarbital anesthesia (40 mg/kg). The lung tissue was collected and either fixed in paraformaldehyde or stored at −80 °C. The Animal Ethics Committee of Jilin Agricultural University approved the animal experiment (Animal Experimentation Ethics Number: 20231206001).

### 2.4. Cell Model Establishment

Primary alveolar macrophages were isolated from rat bronchoalveolar lavage fluid (BALF) in accordance with the methodologies described in the literature [18]. Furthermore, the cells were subjected to CD68 immunofluorescence staining in order to confirm their identity as macrophages. Cells were cultured in high glucose DMEM medium containing 10% FBS and supplemented with 100 U/mL penicillin and 0.1 mg/mL streptomycin (Beyotime Biotechnology, Shanghai, China) at 37 °C within 5% CO_2_ atmosphere.

Cells were divided into Control, PM2.5, 4-PBA, PM2.5 + 4-PBA, Ionomycin, BAPTA-AM, and Ionomycin + BAPTA-AM groups. To investigate the toxic effects of PM2.5 on cells, they were exposed to 20, 60, and 180 μg/mL of PM2.5. The effects of PM2.5 on the cells were then assessed after 12 h of exposure. To investigate the role of ER stress in PM2.5-exposed cells, we established a group treated with an ER stress inhibitor, 4-PBA (1 μM). Additionally, a [Ca^2+^]i disordered group was established by using Ionomycin (0.5 μM, 6 h, MedChemExpress LLC., NJ, USA), and [Ca^2+^]i levels were inhibited with BAPTA-AM (5 μM, 2 h, MedChemExpress LLC., USA) to confirm the impact of [Ca^2+^]i disorder on cells. Untreated cells were used as a Control group. We determined the concentration of ionomycin and BAPTA-AM to be used based on reported studies [19,20].

### 2.5. Histopathological Examination

Following fixation with 4% paraformaldehyde, dehydration with ethanol, immersion in xylene, and paraffin embedding, lung tissue samples were sectioned. Sections were sealed after staining with hematoxylin and eosin. Observation and image acquisition were performed using a microscope (Phenix, Hangzhou, Zhejiang, China). Three samples were selected randomly from each group for histopathological assessment.

### 2.6. TUNEL Assay

TUNEL assay kits (Vazyme International LLC., Nanjing, China) were applied to detect apoptotic cells in the lung sections, following the manufacturer’s instructions. After the labeling of the cell nuclei with DAPI, TUNEL-positive cells were examined under a fluorescence microscope (Olympus, Tokyo, Japan).

### 2.7. Cell Viability

Cell viability was measured using a CCK-8 kit (Beyotime, Shanghai, China) according to the manufacturer’s instructions. Cells were plated in 96-well plates (5000 cells/well) and exposed to PM2.5 stimulation. Absorbance was measured at 450 nm using a microplate photometer (Thermo Fisher Scientific Inc., Waltham, MA, USA).

### 2.8. Cell ATP Assay

Levels of ATP were determined using kits according to the manufacturer’s instructions (Beyotime, Shanghai, China). The cells were lysed using RIPA and the supernatant was collected via centrifugation at 12,000 rpm/min for 5 min. The protein concentration of the supernatant was determined using the BCA method (Beyotime, Shanghai, China). After taking 20 μL of the supernatant mixed with 100 μL of the assay working solution, RLU values were determined by chemiluminescence (Atila Biosystems, Inc., Sunnyvale, CA, USA). Finally, the concentration of ATP in the samples was calculated from the standard curve.

### 2.9. Cytoplasmic Ca^2+^, JC-1 and Mito-SOX Measurement

Briefly, Flou-4 AM, JC-1 and MitoSOX solutions (Shanghai Yisheng Biotechnology Co., Ltd., Shanghai, China) were incubated with cells for 30 min; the cells were then added to Hank’s Balanced Salt Solution (HBSS) for another 25 min. The fluorescence intensity was quantified using a fluorescence microscope (Olympus, Tokyo, Japan) and a fluorescence microplate reader (Spark, Tecan Group Ltd., Zurich, Switzerland).

### 2.10. Transmission Electron Microscopy (TEM)

At the end of the rat alveolar macrophage treatment, the samples were rinsed with PBS. The samples were transferred to Shanghai Jiao Tong University for 1% osmic acid fixation, gradient ethanol dehydration, epoxy resin embedding and sectioning. Finally, after uranium lead double staining, images were taken using TEM (Helios 5 UX, Thermo Fisher Scientific Inc., Waltham, MA, USA).

### 2.11. Immunofluorescence Staining

Immunofluorescence staining was performed on the alveolar macrophage surface protein CD68. Concurrently, immunofluorescence staining was conducted to assess the expression of NLRP3 in cells following treatment. Cells were sequentially fixed with 4% paraformaldehyde for 20 min, 0.3% Triton-X 100-permeabilized for 10 min, and BSA-closed for 1 h at ambient temperature. Subsequently, the cells were incubated at 4 °C overnight with antibodies against CD68 (1:100; A20803; ABclonal, Inc., Boston, MA, USA) or NLRP3 (1:50; A24294; ABclonal, Inc., Boston, MA, USA). Following this, the cells were exposed to CY3-conjugated anti-rabbit IgG (1:100; SA00009-2; Proteintech Group, Inc., Chicago, IL, USA) at ambient temperature for 1 h. After sufficient washing, DAPI was added to label the nuclei. Finally, images were acquired under a fluorescence microscope (Olympus, Tokyo, Japan).

### 2.12. ELISA

The levels of the cytokines IL-1β, IL-18, IL-6, TNF-α, and LDH were determined using ELISA kits according to the manufacturer’s instructions (Jiangsu Meimian Industrial Co., Ltd., Yancheng, Jiangsu, China).

### 2.13. RT-qPCR

RNA was extracted from the treated cells using Trizol (Takara, Kyoto, Japan) reagent following the manufacturer’s instructions. Total RNA was reverse transcribed into cDNA using the Reagent Kit (Takara, Kyoto, Japan). qPCR reactions were performed using TB Green Mix (Takara, Kyoto, Japan). The CT value for the target gene expression was compared to that of the Control group. *GAPDH* was used as a housekeeping control. Relative quantitative analysis was performed using the 2^−ΔΔCT^ method. The primer sequences utilized in the study are as follows:
**Gene****Forward Primer (5′→3′)****Reverse Primer (5′→3′)***ATF6*TTTGGATTTGATGCCTTGGGAGTCCTGTGGACCGAGGAGAAGAGAC*CHOP*CCTCGCTCTCAAGATTCCAGTCTCATTCTCCTGCTCCTTCTCCTTC*GRP78*GGAGGAGGACAAGAAGGAGGATGTTGAATACACCGACGCAGGAATAG*GAPDH*CCTGCACCACCAACTGCTTACATCACGCCACAGCTTTCCA

### 2.14. Western Blotting

RIPA buffer supplemented with protease inhibitors (PMSF, Sangon Biotech, Shanghai, China) was used to extract total proteins from cells incubated on ice. The proteins underwent separation through SDS-PAGE and were subsequently transferred to PVDF membranes (Millipore, Bedford, MA, USA). The membranes were blocked with 5% non-fat milk for 2 h and subsequently incubated with primary antibodies overnight at 4 °C. Secondary antibodies were applied at 25 °C for 2 h. The optical density of the protein bands was detected, and the expression levels of the proteins were evaluated using ECL detection reagents (Monad, Suzhou, China).

### 2.15. Statistical Analysis

All experiments conducted in this study were repeated three times, and the results are presented as means ± SD. GraphPad 8.0.1 (GraphPad Software, Inc., San Diego, CA, USA) was used to visualize graphs and analyze data. Statistical comparisons were made using unpaired Student’s *t*-tests. A single-factor analysis of variance was used to compare data between multiple groups, and the Tukey–Kramer post hoc test was employed. Statistical significance was set at *p* < 0.05.

## 3. Results

### 3.1. 4-PBA Inhibits PM2.5-Induced Lung Injury in Rats

The chemical structure of 4-PBA is shown in Figure 1C. PM2.5 concentrations fluctuated between 93 and 249 μg/m^3^ over a 24 h period based on measurements taken within the target environment. The daily average concentration was 186.3 μg/m^3^, as illustrated in Figure 1D. The W/D ratio (wet/dry ratio, W/D) of lung injuries was significantly higher in PM2.5-exposed rats compared to the Control group. Pretreatment with 4-PBA significantly reduced the increase in the W/D ratio (Figure 1E). Pretreatment with 4-PBA significantly reduced the number of white blood cells, lymphocytes and granulocytes in the blood compared to the PM2.5 group (Table S1). H&E staining of lung sections showed normal lung tissue structure in both the Control and 4-PBA groups. In the PM2.5 group, the lung tissue exhibited significant pathological damage, such as alveolar hemorrhage, alveolar wall thickening, inflammatory cell infiltration, and interstitial alveolar exudates. Pretreatment with 4-PBA protected against the pathological changes induced by PM2.5 (Figure 1F,G). The results suggest that pretreatment with 4-PBA effectively reduced lung injury induced by PM2.5.

### 3.2. 4-PBA Inhibits PM2.5 Exposure-Induced ER Stress in Rat Lungs

**Figure 2:**
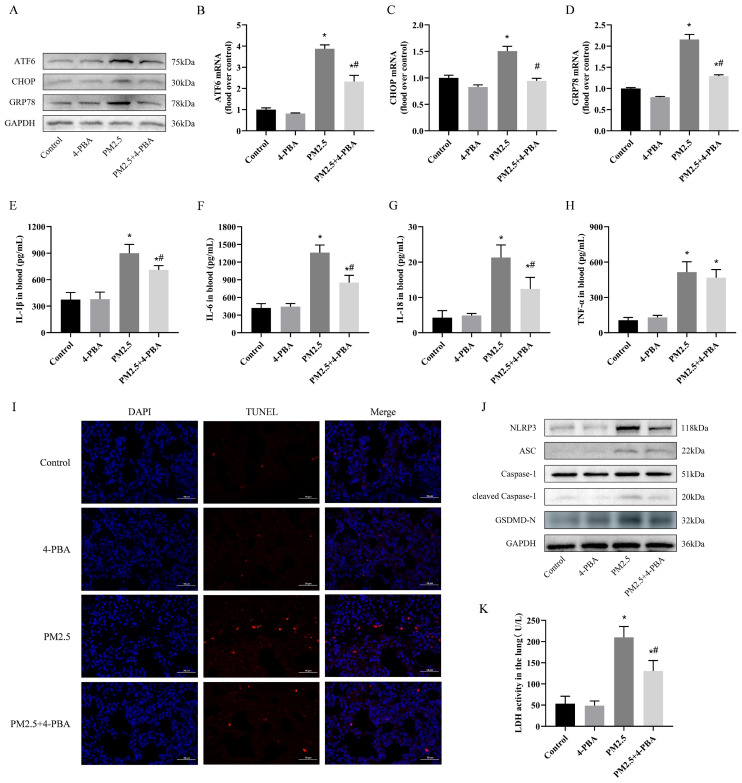
4-PBA inhibits lung ER stress, inflammation and pyroptosis induced by PM2.5 in rats. (**A**) Detection of ATF6, CHOP and GRP78 expression via Western blotting after treatment with PM2.5 and pretreatment with 4-PBA. (**B**–**D**) Detection of *ATF6*, *CHOP* and *GRP78* mRNA expression via qRT-RCR. (**E**–**H**) The levels of IL-1β, IL-6, IL-18 and TNF-α expression in the blood after treatment with PM2.5 and 4-PBA. (**I**) TUNEL staining of rat lung tissue sections (red). Scale bar = 50 μm. (**J**) Detection of NLRP3, ASC, Caspase-1, cleaved Caspase-1 and GSDMD-N expression via Western blotting after treatment with PM2.5 and pretreatment with 4-PBA. (**K**) LDH activity in the lungs of rats. Results are expressed as the mean ± SD of triplicate determinations. * *p* < 0.05, compared with the Control group; ^#^ *p* < 0.05, compared with the PM2.5 group. Original images of (**A**,**J**) can be found in Supplementary Materials.

To assess whether the inhibitory effect of 4-PBA on lung injury induced by PM2.5 is related to endoplasmic reticulum stress, we evaluated the level of ER stress in rat lungs. As shown in Figure 2A–D, *ATF6*, *CHOP* and *GRP78* mRNA and protein expression were significantly increased in lung tissue after PM2.5 exposure compared to the Control group, and 4-PBA pretreatment was targeted toward inhibiting the onset of ER stress and reversing the above changes. The above results indicated that PM2.5 effectively induced ER stress in rat lungs, and the inhibitory effect of 4-PBA on PM2.5-induced lung injury might be related to ER stress.

### 3.3. 4-PBA Inhibits Lung Inflammation and Pyroptosis Induced by PM2.5 in Rats

In the PM2.5 group, the levels of IL-1β, IL-6, IL-18, and TNF-α were significantly higher than those in the Control group, and 4-PBA pretreatment significantly reduced the increase in these pro-inflammatory factors (Figure 2E–H). Pretreatment with 4-PBA significantly reduced the TUNEL apoptotic index of lung tissue caused by exposure to PM2.5, as shown in Figure 2I. Furthermore, Western blot analysis revealed a significant increase in the protein levels of NLRP3, ASC, Caspase-1 and GSDMD-N following exposure to PM2.5. LDH (lactate dehydrogenase) levels were significantly elevated in rat lungs under the influence of PM2.5 exposure. 4-PBA was similarly found to attenuate the expression of these signals in the lungs. (Figure 2J,K). These findings indicate that 4-PBA can inhibit lung pyroptosis and inflammation caused by exposure to PM2.5.

### 3.4. PM2.5 Induces Damage to Rat Alveolar Macrophages In Vitro

**Figure 3:**
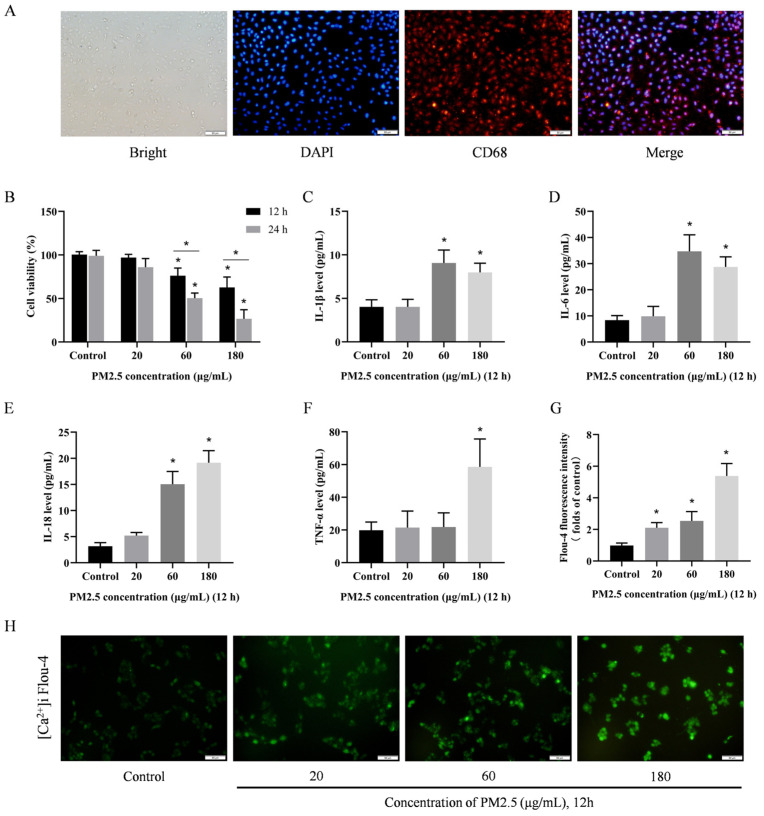
PM2.5 induces damage to rat alveolar macrophages in vitro. (**A**) CD68 immunofluorescence staining of primary rat alveolar macrophages. Scale bar = 50 μm. (**B**) CCK-8 assay showing the toxicity of cells exposed to PM2.5. (**C**–**F**) The levels of IL-1β, IL-6, IL-18 and TNF-α expression in the cells after PM2.5 treatment. (**G**) Fluo-4 AM staining, cell fluorescence intensity was measured using a fluorescence microplate reader. (**H**) Fluo-4 AM staining after PM2.5 treatment. Scale bar = 50 μm. Results are expressed as the mean ± SD of triplicate determinations. * *p* < 0.05, compared with the Control group.

To investigate the mechanism behind the lung toxicity caused by PM2.5, an exposure model was constructed using rat alveolar macrophages in vitro. Figure 3A illustrates the CD68 immunofluorescence staining of rat primary alveolar macrophages, demonstrating that the alveolar macrophages extracted from BALF were of high purity and met the requisite standards for in vitro studies. The cells were exposed to PM2.5 concentrations of 20, 60, and 180 μg/mL. After 12 and 24 h of exposure, low and intermediate concentrations of PM2.5 had no significant effect on cell viability, whereas high concentrations of PM2.5 significantly reduced cell viability (Figure 3B). Similarly, the expression of IL-1β, IL-6, IL-18, and TNF-α was significantly increased in a dose-dependent manner after 12 h of exposure (Figure 3C–F). Notably PM2.5 markedly raised [Ca^2+^]i levels; low concentrations of PM2.5 were sufficient to trigger this quick reaction (Figure 3G,H). These findings imply that considerable alveolar macrophage damage could be induced by PM2.5.

### 3.5. PM2.5-Induced Rat Alveolar Macrophage ER Stress and Mitochondrial Damage

**Figure 4:**
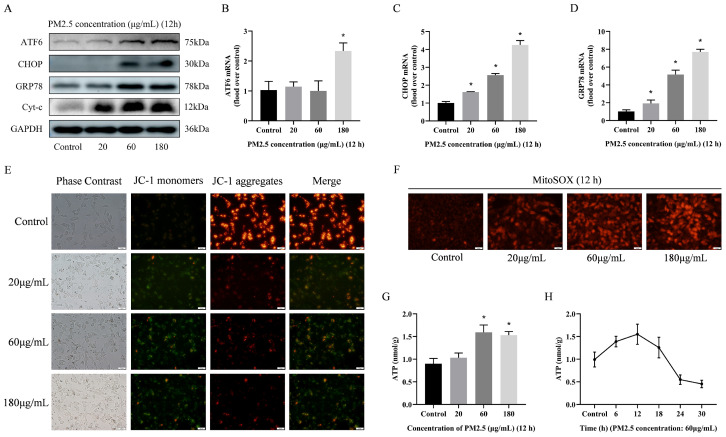
PM2.5-induced rat alveolar macrophage ER stress and mitochondrial damage. (**A**) Detection of ATF6, CHOP, GRP78 and Cyt-c expression via Western blotting after PM2.5 treatment. (**B**–**D**) Detection of *ATF6*, *CHOP* and *GRP78* mRNA expression via qRT-RCR. (**E**) Fluorescent staining of cellular mitochondrial membrane potential JC-1. Scale bar = 20 μm. (**F**) Fluorescence staining of cellular mitochondrial superoxide MitoSOX. Scale bar = 20 μm. (**G**,**H**) Changes in cellular ATP levels after PM2.5 treatment. Results are expressed as the mean ± SD of triplicate determinations. * *p* < 0.05, compared with the Control group;. Original images of (**A**) can be found in Supplementary Materials.

Due to the crucial role of Ca^2+^ in cellular physiological processes, it is imperative to investigate the causes of PM2.5-induced [Ca^2+^]i disorders. This study therefore focused on two important intracellular calcium reservoirs (the endoplasmic reticulum and mitochondria). The effects of PM2.5 on these organelles were assessed. As shown in Figure 4A–D, the mRNA and protein levels of ATF6, CHOP, and GRP78 were increased after exposure to PM2.5. Similarly, the protein expression of Cyt-c was increased, and the mitochondrial membrane potential of JC-1 significantly decreased, but the level of mitochondrial superoxide MitoSOX significantly increased (Figure 4E,F). Interestingly, after PM2.5 stimulation, the cellular ATP levels significantly increased within 12 h, but showed a sudden decreasing trend over time (Figure 4G,H). These results suggest that PM2.5 exposure significantly induces damage to the calcium pool of alveolar macrophages in vitro. PM2.5-induced [Ca^2+^]i disruption can be associated with damage to the intracellular calcium pool.

### 3.6. Inhibition of ER Stress by 4-PBA Attenuated PM2.5-Induced [Ca^2+^]i Disorders and Alterations in MAMs

**Figure 5:**
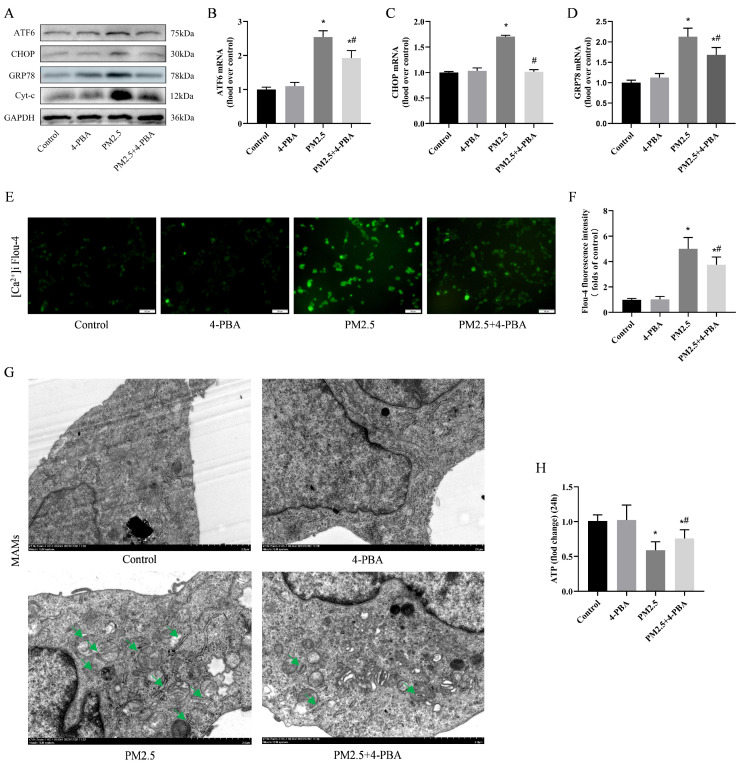
Inhibition of ER stress by 4-PBA attenuated PM2.5-induced [Ca^2+^]i disorders and alterations in MAMs. (**A**) Detection of ATF6, CHOP, GRP78 and Cyt-c expression via Western blotting after treatment with PM2.5 and pretreatment with 4-PBA. (**B**–**D**) Detection of ATF6, CHOP and GRP78 mRNA expression via qRT-RCR. (**E**) Fluo-4 AM staining after treatment with PM2.5 and pre-treatment with 4-PBA. Scale bar = 50 μm. (**F**) Fluo-4 AM staining cell fluorescence intensity was measured using a fluorescence microplate reader. (**G**) A change in the number of cellular MAM structures was observed in TEM images (green arrows). Scale bar = 2 μm. (**H**) Changes in cellular ATP levels after treatment with PM2.5 and pre-treatment with 4-PBA. Results are expressed as the mean ± SD of triplicate determinations. * *p* < 0.05, compared with the Control group; ^#^ *p* < 0.05, compared with the PM2.5 group. Original images of (**A**) can be found in Supplementary Materials.

To investigate whether endoplasmic reticulum stress is associated with [Ca^2+^]i disorders, we used 4-PBA pretreatment (1 μM, 6 h). Similar to in vivo, 4-PBA inhibited the expression of ATF6, CHOP, and GRP78 induced by PM2.5 (Figure 5A–D), while partially restoring [Ca^2+^]i levels (Figure 5E,F). Mitochondria-associated endoplasmic reticulum membrane changes (MAMs) were used to measure endoplasmic reticulum and mitochondrial crosstalk. The TEM results show that MAMs increased significantly after PM2.5 exposure, and 4-PBA effectively suppressed the changes in MAMs (Figure 5G). Furthermore, 4-PBA has an inhibitory effect on the expression of Cyt-c (Figure 5A), and a restoring effect on ATP levels after 24 h of PM2.5 stimulation in cells (Figure 5H). This indicates that PM2.5-induced disruption of [Ca^2+^]i is associated with ER stress and affects mitochondrial function to some extent through MAMs.

### 3.7. 4-PBA Inhibited PM2.5-Induced Cellular Inflammation and Pyroptosis

**Figure 6:**
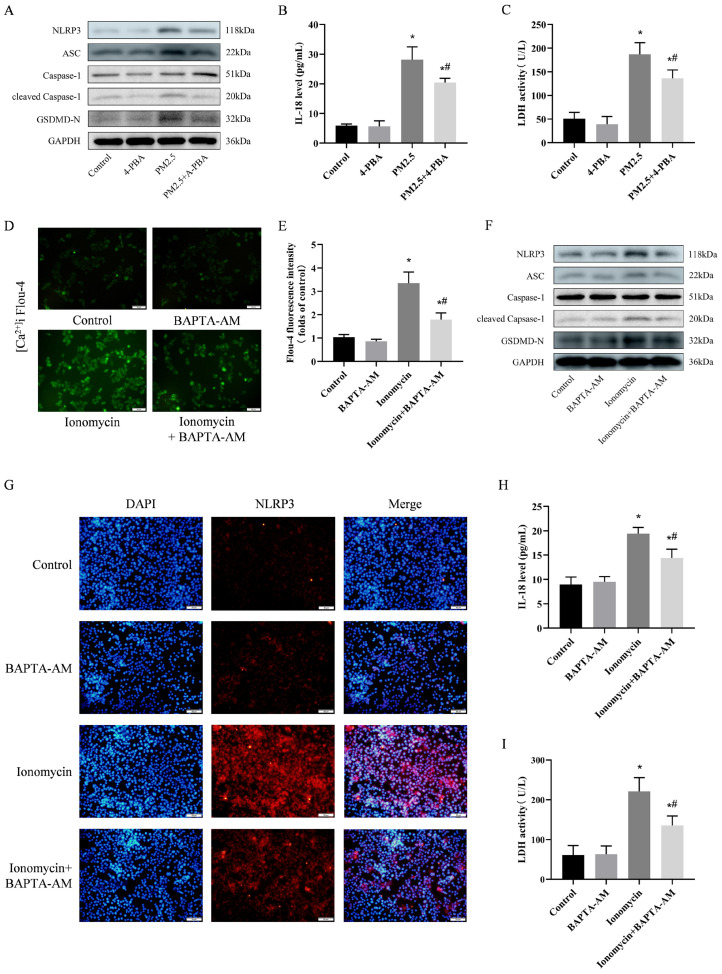
Preventing [Ca^2+^]i disorder is an important pathway to weaken NLRP3-mediated pyroptosis. (**A**) Detection of NLRP3, ASC, Caspase-1, cleaved Caspase-1 and GSDMD-N expression by Western blotting after treatment with PM2.5 and pre-treatment with 4-PBA. (**B**,**H**) Levels of IL-18 expression in cells. (**C**,**I**) LDH activity in cells. (**D**) Fluo-4 AM staining after treatment with ionomycin and pre-treatment with BAPTA-AM. Scale bar = 50 μm. (**E**) Fluo-4 AM staining cell fluorescence intensity was measured using a fluorescence microplate reader. (**F**) Detection of NLRP3, ASC, Caspase-1, cleaved Caspase-1 and GSDMD-N expression via Western blotting after treatment with ionomycin and pre-treatment with BAPTA-AM. (**G**) NLRP3 immunofluorescence staining of cells after treatment with ionomycin and pre-treatment with BAPTA-AM. Scale bar = 50 μm. Results are expressed as the mean ± SD of triplicate determinations. * *p* < 0.05, compared with the Control group; ^#^ *p* < 0.05, compared with the PM2.5 group. Original images of (**A,F**) can be found in Supplementary Materials.

To further explore the intrinsic link between PM2.5-induced cellular damage and ER stress, we detected the expression of NLRP3. As shown in Figure 6A, PM2.5 significantly promoted the expression of NLRP3, ASC and cleaved-Caspase-1. 4-PBA pretreatment was able to inhibit the expression of NLRP3. This suggests that 4-PBA may inhibit the inflammatory response by affecting the expression of NLRP3, which serves as an important factor in the occurrence of cellular pyroptosis. We also measured the onset of cellular pyroptosis. The expression of GSDMD-N, IL-18 and LDH was also reduced by 4-PBA (Figure 6A–C). This is consistent with the results of the in vivo experiments. This suggests that 4-PBA is able to attenuate the cellular inflammatory response and cellular pyroptosis by inhibiting endoplasmic reticulum stress.

### 3.8. Preventing [Ca^2+^]i Disorder is an Important Pathway in Weakening NLRP3-Mediated Pyroptosis

According to the above results, 4-PBA showed a controlling effect on ER stress via Ca^2+^ under the influence of PM2.5. Therefore, to investigate the effects of [Ca^2+^]i disruption after ER stress, we used ionomycin to mimic intracellular Ca^2+^ and an intracellular Ca^2+^ chelator (BAPTA-AM) to inhibit [Ca^2+^]i levels. The results showed that pretreatment with BAPTA-AM effectively reversed ionomycin-induced [Ca^2+^]i disorder (Figure 6D,E), ionomycin significantly induced the expression of NLRP3 and its downstream molecules, and the [Ca^2+^]i chelator BAPTA-AM inhibited the elevation in NLRP3, LDH, and IL-18 (Figure 6F–I). This indicates that when cells are subjected to adverse stimuli that induce [Ca^2+^]i disorder, inflammation and death are promoted. This mechanism is closely related to NLRP3 activation-mediated cell pyroptosis.

## 4. Discussion

The results from monitoring changes in PM2.5 concentrations in a cattle farm environment indicate that the PM2.5 concentrations far exceeded the prescribed standard concentration limits (the WHO recommended a daily limit of 75 μg/m^3^) [21]. This means that in large and medium-sized animal breeding facilities, due to various reasons such as frequent human and animal activities and the operation of large machinery, PM2.5 has become a significant potential threat to respiratory health [22]. Concurrently, this may be a significant contributing factor to the susceptibility of animals to respiratory infections. There is a substantial body of evidence indicating that PM2.5 has the capacity to promote lung inflammation and associated cell death [23,24]. In particular, PM2.5 has been demonstrated to induce a number of different types of cell death, including autophagy, necrosis, apoptosis, pyroptosis, and ferroptosis [25,26]. Although the sources of PM2.5 in these studies were disparate, they all reported comparable biological toxicities. Our study demonstrated a correlation between pulmonary injury and in vitro cell death in rats following exposure to PM2.5. The lungs of rats exposed to cowshed PM2.5 exhibited alveolar destruction, septal thickening, inflammatory cell infiltration, and a massive release of inflammatory cytokines. Additionally, an increase in a variety of inflammatory cells, including neutrophils, lymphocytes, and eosinophils, was observed in the blood. This is comparable to the findings of numerous respiratory toxicity studies conducted on urban PM2.5 [27,28]. The data indicate that the lung inflammation and structural damage caused by PM2.5 exposure do not differ according to the source of exposure. A number of aromatic hydrocarbon compounds present in urban PM2.5 have been identified as playing a significant role in the development of respiratory damage [29]. Due to the particularities of the cowshed environment, in our previous research, we not only conducted a comprehensive analysis of the chemical composition of PM2.5 in cowsheds but also focused on the composition of microbial components [4]. In addition to containing a significant number of aromatic compounds, the abundance of microorganisms might be a significant contributing factor in the exacerbation of respiratory toxicity associated with PM2.5.

The rats in this study were exposed to PM2.5 for 30 days, and the damage to the organism from prolonged exposure to PM2.5 may not be limited to the respiratory system. Long-term exposure to PM2.5 has been found to be associated with increased blood pressure, increased heart rate and the induction of serious cardiovascular events such as myocardial infarction and heart failure [30]. Prolonged exposure to PM2.5 can affect the functioning of the immune system and worsen the condition of patients with immune system disorders [31]. Furthermore, the rats in this study were of a similar age and body weight, and it is possible that performance may vary depending on age in the presence of PM2.5 exposure. The respiratory and immune systems are not yet fully developed in early childhood, rendering them more susceptible to the effects of PM2.5. Long-term exposure may result in an increased susceptibility to respiratory infections, a higher frequency of asthma attacks, and disruption to the normal development of lung function [32]. As individuals age, there is a natural decline in bodily functions, particularly in the respiratory and cardiovascular systems. Prolonged exposure to PM2.5 may exacerbate existing conditions such as emphysema and heart disease, and in some cases, may even lead to a deterioration in overall health. The likelihood of infection is increased in older age groups, where immune function is diminished and the capacity to resist and eliminate pollutants is reduced [33]. Furthermore, prolonged exposure to PM2.5 during pregnancy has been linked to adverse effects on the fetus. A number of studies have demonstrated that exposure to PM2.5 can result in morphological alterations in ovarian tissues, fluctuations in hormone levels, and a reduction in fertility in experimental animals, and in vitro cellular experiments have corroborated these findings, indicating that PM2.5 induces oxidative stress, inflammatory responses and apoptosis in ovarian cells [34]. In the future, more accurate exposure assessment methods should be used in order to facilitate further research into the molecular mechanisms of damage induced by PM2.5.

In this study, to investigate the causes of PM2.5 damage to lungs and cells, we focused on the effects of PM2.5 on two organelles: the endoplasmic reticulum and the mitochondria. The data indicate that exposure to the cowshed PM2.5 induces widespread endoplasmic reticulum stress and mitochondrial damage. A number of studies have demonstrated that the polycyclic aromatic hydrocarbons present in PM2.5 can induce mitochondrial damage and ER stress, activate multiple pathways such as MAPK, PI3K/Akt, and JAK/STAT signaling, and induce cell death [35,36]. In addition, PM2.5-induced ROS and oxidative stress are important causes of mitochondrial damage [37]. A previous study conducted by our research group similarly demonstrated the significance of oxidative stress [38]. However, at this point, it is difficult to determine the true role of endoplasmic reticulum stress in this section, since it can be viewed as a double-edged sword. It has been demonstrated that moderate and timely ER stress can contribute to proper refolding, thereby alleviating cellular stress and improving survival conditions. However, excessive ER stress has been identified as a key initiator in the process of pyroptosis [39]. Our data show elevated levels of IL-1β and IL-18 expression in rat lung tissue. We reasoned that pyroptosis may be involved in PM2.5-induced inflammation and lung injury. Pyroptosis is a type of programmed cell death that is induced by inflammatory vesicles. In the context of inflammatory vesicles, NLRP3 plays a pivotal role in the recognition of pathogen-associated molecular patterns or injury-associated molecular patterns [40]. The activation of NLRP3 inflammatory vesicles resulted in the cleavage of Caspase-1, which in turn led to an increase in the expression of GSDMD-N, IL-1β, and IL-18 [41]. This process ultimately resulted in pyroptosis. The results of this study provide evidence that cowshed PM2.5 plays a role in initiating pyroptosis.

In order to address the point that PM2.5 induces ER stress and mitochondrial dysfunction, some studies have demonstrated that by regulating key factors in the pathway related to ER stress and mitochondrial damage, such as the inhibition of PERK, IRE1α, or BTK, downstream signaling can be reduced, thereby alleviating cellular damage [42,43,44]. Molecular inhibitors have demonstrated some efficacy in animal models and cellular experiments. However, due to the complexity of the signaling pathways, there is still a need for further validation in clinical applications. ER stress and mitochondrial dysfunction are closely associated with oxidative stress. The administration of antioxidants, such as vitamin B and N-acetylcysteine (NAC), has been demonstrated to attenuate these manifestations [45,46]. The safety of vitamins and antioxidants has been established through clinical use, yet further investigation is required to ascertain their precise impact on the endoplasmic reticulum and mitochondria. It is notable that our study demonstrated that during PM2.5-induced ER stress and mitochondrial damage, alterations in [Ca^2+^]i may act as an intermediary mediator between these two organelles. In previous studies, it was observed that PM2.5 induced changes in G proteins (Gq/11), which in turn led to the massive expression of the IP_3_R (Inositol 1,4,5-trisphosphate (IP3) receptor). The IP_3_R channel is a crucial regulator of the [Ca^2+^]i environment within the endoplasmic reticulum. An imbalance in this channel can result in the release of excessive [Ca^2+^]i [5]. In this study, following the stimulation of cells by PM2.5, the number of MAM structures increased in the cells, and the mitochondria received excess Ca^2+^. This resulted in the mitochondria becoming overworked by stimulation, leading to the enlargement of the cell’s mitochondria and dysfunction. The data obtained support this conclusion. In particular, the mitochondrial ATP processing is abnormal. After receiving PM2.5 stimulation, the cellular ATP level rapidly increases in a short period of time and then drops sharply. Recent studies have shown that under physiological conditions, cellular ATP levels are influenced by various factors, such as oxidative stress, calcium overload, hypoxia, and increased mitochondrial membrane permeability, which promote the compensatory synthesis of mitochondrial ATP. This is manifested as a transient increase in ATP levels, which constitutes the source signal of metabolic disorders [47,48]. The above process is similar to the results for ATP level changes in this study. 4-PBA has been widely shown to inhibit ER stress [49]. In our study, 4-PBA exerted a significant protective effect during lung injury and cellular damage in rats, attenuating PM2.5-induced airway inflammation and pyroptosis. The results demonstrated that pretreatment with 4-PBA resulted in a reduction in ER stress, accompanied by a restoration of [Ca^2+^]i levels and mitochondrial damage. Furthermore, the cytotoxicity induced by PM2.5 was found to be attenuated, as was the gene transcription associated with pyroptosis. The above findings demonstrate that the triad of the endoplasmic reticulum, [Ca^2+^]i, and mitochondria are inextricably linked, which represents an important addition to our previous studies. Furthermore, the data indicate that pyroptosis is a significant effector process following the stimulation of an imbalance in the endoplasmic reticulum, mitochondrial function and [Ca^2+^]i by PM2.5. Consequently, the maintenance of [Ca^2+^]i homeostasis and mitochondrial function by maintaining endoplasmic reticulum function represents an important therapeutic option for treating PM2.5-induced lung injury.

Disruptions in [Ca^2+^]i are not exclusively caused by endoplasmic reticulum and mitochondrial dysfunction. After mitochondria and the endoplasmic reticulum, lysosomes were discovered as a new cellular calcium pool. Once the permeability of the lysosomal membranes changes, a large amount of Ca^2+^ and various hydrolytic enzymes are released into the cytoplasm, usually leading to cell death, known as lysosome-dependent cell death [50]. Although there are relatively few reports on this, it may be another potential reason for PM2.5-induced cell death. Ca^2+^ has been shown to boost intracellular Ca^2+^ concentrations in a variety of ways, in addition to being released from intracellular calcium reserves. When cells are stimulated by PM2.5, voltage- or ligand-gated channels located on the cell membrane can also mediate extracellular Ca^2+^ influx and further activate NLRP3 [51]. On the other hand, the ways of regulating a [Ca^2+^]i steady state are also diverse. In this study, BAPTA-AM was found to be an effective inhibitor of [Ca^2+^]i overload. However, this approach is not widely applicable in the real environment due to the disruptive effect of BAPTA-AM on the negative feedback regulation process after the cellular [Ca^2+^]i level has returned to a normal range. As previously observed, the administration of BAPTA-AM in excess of the optimal dosage results in cytotoxic effects. Furthermore, the regulation of [Ca^2+^]i by RGS2 (Regulator of G-protein Signaling 2) is achieved through the mediation of G-protein signaling, which is comparatively less intense than BAPTA-AM and does not disrupt the negative feedback process of cellular Ca^2+^ signaling [5]. Similarly, we used 4-PBA to inhibit ER stress to regulate [Ca^2+^]i in the present study, again in a relatively mild manner, with the endoplasmic reticulum and mitochondria making adaptive changes in response to [Ca^2+^]i levels. Compared to BAPTA-AM, the application of 4-PBA may have more clinical and therapeutic significance. This study demonstrated the relationship between [Ca^2+^]i disorders and PM2.5-induced lung injury. However, the damage caused by PM2.5 is not limited to the lungs, and many reports indicate that PM2.5 also has a damaging effect on the cardiovascular, nervous, and reproductive systems [52,53,54]. Whether [Ca^2+^]i disorders have a similar role in PM2.5-induced diseases in other systems is unknown, and this demonstrates the limitations of our current study. Consequently, the mechanism behind [Ca^2+^]i action in PM2.5-induced damage in various systems will be the focus of our future research.

## 5. Conclusions

In summary, our study demonstrated that the use of 4-PBA to target ER stress effectively attenuated PM2.5-induced rat lung injury and rat alveolar macrophage injury. 4-PBA was found to inhibit the effects of the PM2.5 present in cowsheds on ER stress, [Ca^2+^]i disorders, MAMs, abnormal mitochondrial function, and pyroptosis. The findings of this study provide valuable insights into the relationship between PM2.5-induced ER stress, [Ca^2+^]i disruption, mitochondrial damage, inflammation, and pyroptosis. 4-PBA may represent a promising clinical drug in the prevention and treatment of PM2.5-induced respiratory diseases.

## Data Availability

The original contributions presented in the study are included in the article/supplementary material, further inquiries can be directed to the corresponding authors.

## Supplementary Materials

The following supporting information can be downloaded at: https://www.mdpi.com/article/10.3390/biom14091135/s1, Original images of Figure 2A,J, Figure 4A, Figure 5A and Figure 6A,F can be found in Supplementary Materials. Table S1. Complete blood count of rats.
