# Supplementary material for: Attenuation of PM2.5-Induced Lung Injury by 4-Phenylbutyric Acid: Maintenance of [Ca2+]i Stability between Endoplasmic Reticulum and Mitochondria
Source: Biomolecules. 2024 Sep 8;14(9):1135. doi: 10.3390/biom14091135 (PMC11430257; doi:10.3390/biom14091135)
Supplement: Supplementary file 1 [file biomolecules-14-01135-s001.zip › 3. Ethical approval.pdf]

Attenuation of PM2.5-induced lung injury by 4-phenylbutyric acid: maintenance of [Ca^{2+}]i stability between endoplasmic reticulum and mitochondria

2.1. Ethical approval

The experimental animal standard schemes used in this study follow the JLAU Animal Experiment Regulations (JLAU08201409). Every step of this study's procedure follows the ARRIVE guidelines (https://arriveguidelines.org) and the National Research Council's Guide for the Care and Use of Laboratory Animals. All animal experiments were approved by the Laboratory Animal Welfare and Ethics Committee of Jilin Agricultural University (approval code 20231206001).

Ethic Committee Name: Laboratory Animal Welfare and Ethics Committee of Jilin Agricultural University

Project identification code: 20231206001

Approval Date: December 6, 2023
