# Supplementary material for: Attenuation of PM2.5-Induced Lung Injury by 4-Phenylbutyric Acid: Maintenance of [Ca2+]i Stability between Endoplasmic Reticulum and Mitochondria
Source: Biomolecules. 2024 Sep 8;14(9):1135. doi: 10.3390/biom14091135 (PMC11430257; doi:10.3390/biom14091135)
Supplement: Supplementary file 1 [file biomolecules-14-01135-s001.zip › CVs.pdf]

CVs:
Zhenhua Ma: College of Animal Science and Technology, Jilin Agricultural University. Current PhD student in veterinary medicine. Main research interests: animal disease prevention and environmental control.
Xiaohui Du: College of Animal Science and Technology, Jilin Agricultural University. Master's Degree in Veterinary Medicine.
Yize Sun: College of Animal Science and Technology, Jilin Agricultural University. Master's Degree in Veterinary Medicine.
Yunna Jia: College of Animal Science and Technology, Jilin Agricultural University. Current PhD student in veterinary medicine.
Xiaojun Liang: Institute of Animal Science, Ningxia Academy of Agriculture and Forestry Sciences. Doctor of Veterinary Medicine. Professor.
Yunhang Gao: College of Animal Science and Technology, Jilin Agricultural University. Doctor of Veterinary Medicine. Professor. Main research interests: animal disease prevention and environmental control.
