# Supplementary material for: Attenuation of PM2.5-Induced Lung Injury by 4-Phenylbutyric Acid: Maintenance of [Ca2+]i Stability between Endoplasmic Reticulum and Mitochondria
Source: Biomolecules. 2024 Sep 8;14(9):1135. doi: 10.3390/biom14091135 (PMC11430257; doi:10.3390/biom14091135)
Supplement: Supplementary file 1 [file biomolecules-14-01135-s001.zip › Supplemental data about Table S1.pdf]

Table S1

Complete blood count of rats.

	Control			4-PBA			PM2.5			PM2.5+4-PBA		
	1	2	3	1	2	3	1	2	3	1	2	3
White blood cell (WBC) ($\times10^9$/L)	4.5	6.2	5.5	5.4	6.0	4.9	8.9	8.3	7.6	7.3	6.9	8.1
Monocyte count (Mon) ($\times10^9$/L)	0.1	0.1	0.1	0.1	0.2	0.1	0.2	0.3	0.1	0.2	0.1	0.1
Lymphocyte count (Lymph) ($\times10^9$/L)	5.5	5.4	4.9	4.7	6.2	5.1	3.9	4.7	4.2	3.7	3.6	4.5
Granulocyte (Gran) ($\times10^9$/L)	0.8	0.6	0.6	0.6	0.8	0.7	3.5	3.7	2.9	1.3	3.1	3.2
Lymphocyte ratio (%)	77.9	87.3	85.3	87.5	86.3	88.8	60.2	53.4	56.3	60.3	66.7	80.9
Monocytes ratio (%)	2.4	1.5	2.9	1.8	2.6	2.2	2.6	3.2	3	1.9	2.8	2.4
Granulocyte ratio (%)	21.5	13.3	15.2	10.9	11.1	20.3	39.8	45.1	29.9	23.9	27.5	35.1
Red blood cell (RBC) ($\times10^{12}$/L)	7.86	7.66	6.33	7.02	7.9	6.48	6.89	7.2	6.78	7.09	6.37	7.11

	Control			4-PBA			PM2.5			PM2.5+4-PBA		
	1	2	3	1	2	3	1	2	3	1	2	3
Hemoglobin (HGB) (g/L)	144	159	163	177	153	185	177	165	196	133	129	177
Hematocrit (HCT) (%)	43.6	48.3	40.8	53.2	47.2	55	53	49	47	50	45.1	48.3
Mean corpuscular volume (MCV) (fL)	65.5	64	60.1	66.1	62	60	59	61	63.3	62.4	62.8	66.1
Mean corpuscular hemoglobin (MCH) (pg)	23	22.9	23	22.8	23	23.1	22.2	23.9	22.8	23.1	23.2	23.7
Mean corpuscular hemoglobin concentration (MCHC) (g/L)	333	365	384	374	395	360	350	355	369	370	348	367
Red blood cell distribution width (RDW) (%)	9.3	8.7	8.5	8.8	8.2	9.5	9.9	11	10.6	10.9	10.4	9.8
Platelet count (PLT) ($\times 10^9$/L)	868	1080	1366	1265	980	1060	884	1356	986	1155	1032	1196

	Control			4-PBA			PM2.5			PM2.5+4-PBA		
	1	2	3	1	2	3	1	2	3	1	2	3
Mean platelet volume (MPV) (fL)	6.9	7.1	6.6	6.9	6.5	6.6	6.6	6.7	6.6	6.9	6.7	7.1
Platelet distribution width (PDW)	15.2	16	15.2	15.5	15.5	16	15.5	15.5	16	15.9	15.8	15.5
Platelet hematocrit (PCT) (%)	0.512	***	0.533	***	0.561	***	0.511	***	0.564	***	0.502	***

Notes: The symbol * * * indicates that the item in question was not detected.
