# Supplementary figures and images for: Attenuation of PM2.5-Induced Lung Injury by 4-Phenylbutyric Acid: Maintenance of [Ca2+]i Stability between Endoplasmic Reticulum and Mitochondria
Source: Biomolecules. 2024 Sep 8;14(9):1135. doi: 10.3390/biom14091135 (PMC11430257; doi:10.3390/biom14091135)

Supplemental data about western blots

Fig. 2F

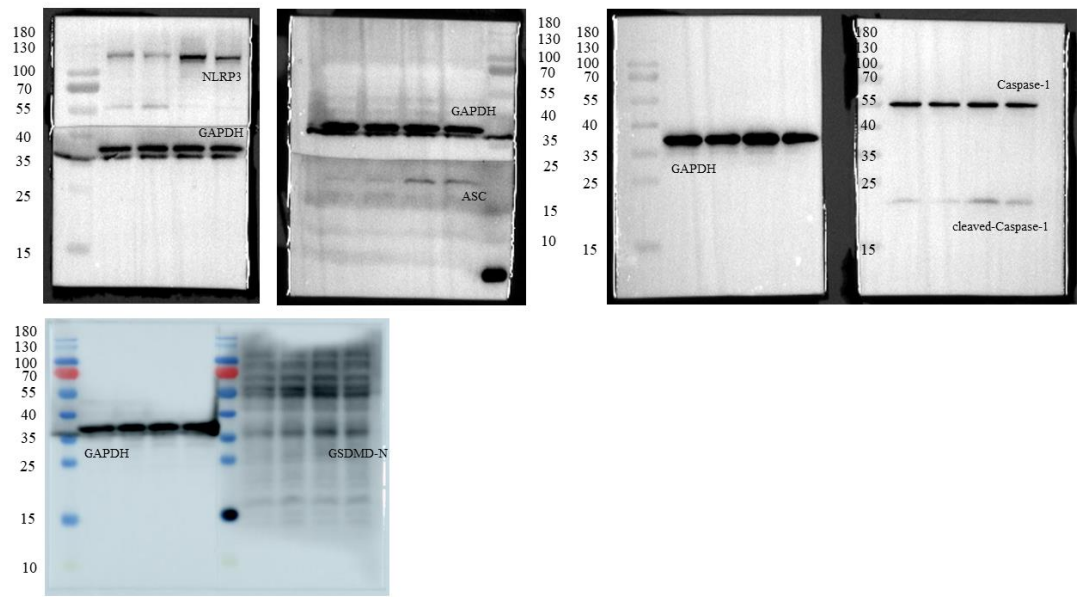

Fig. 2H

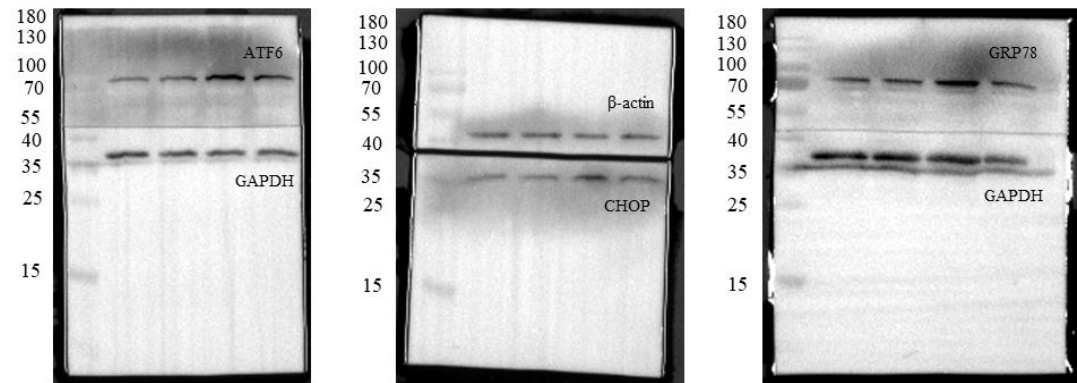

Fig. 4A

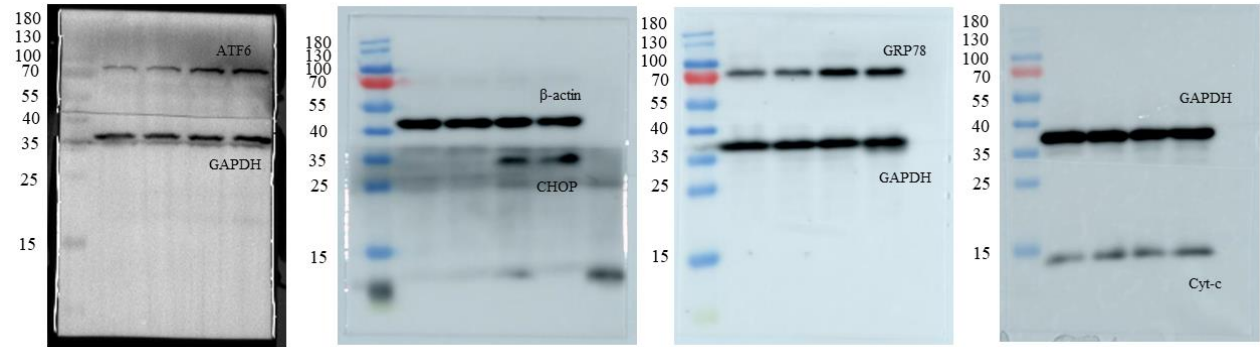

Fig. 5A

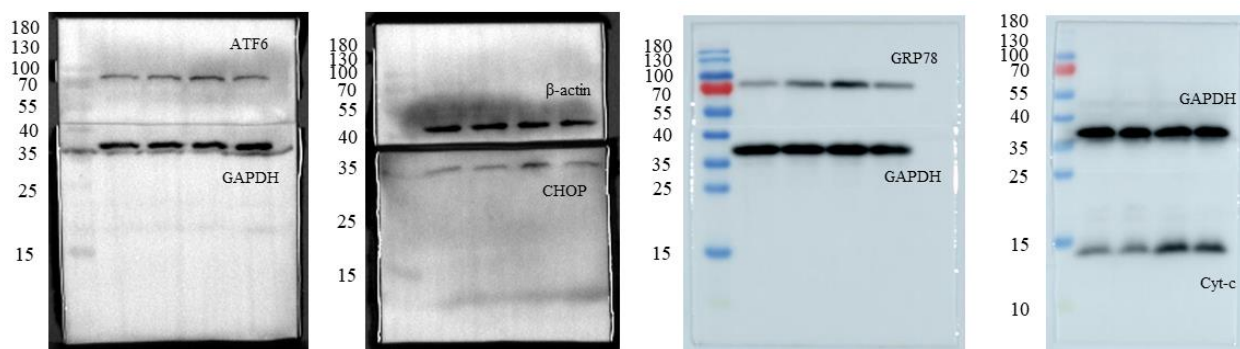

Fig. 6A

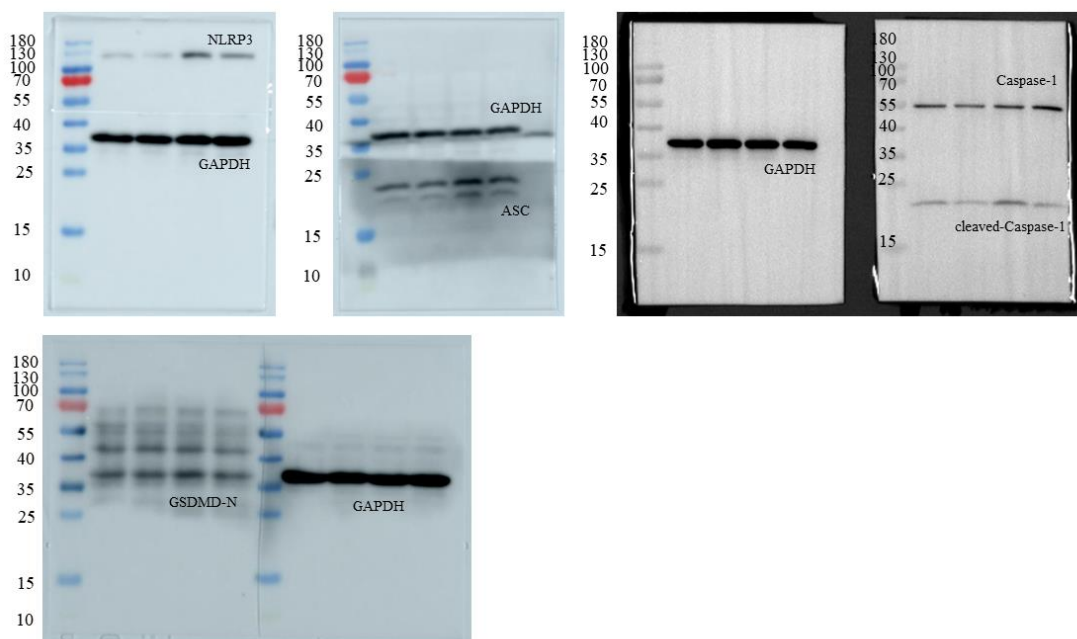

Fig. 6F

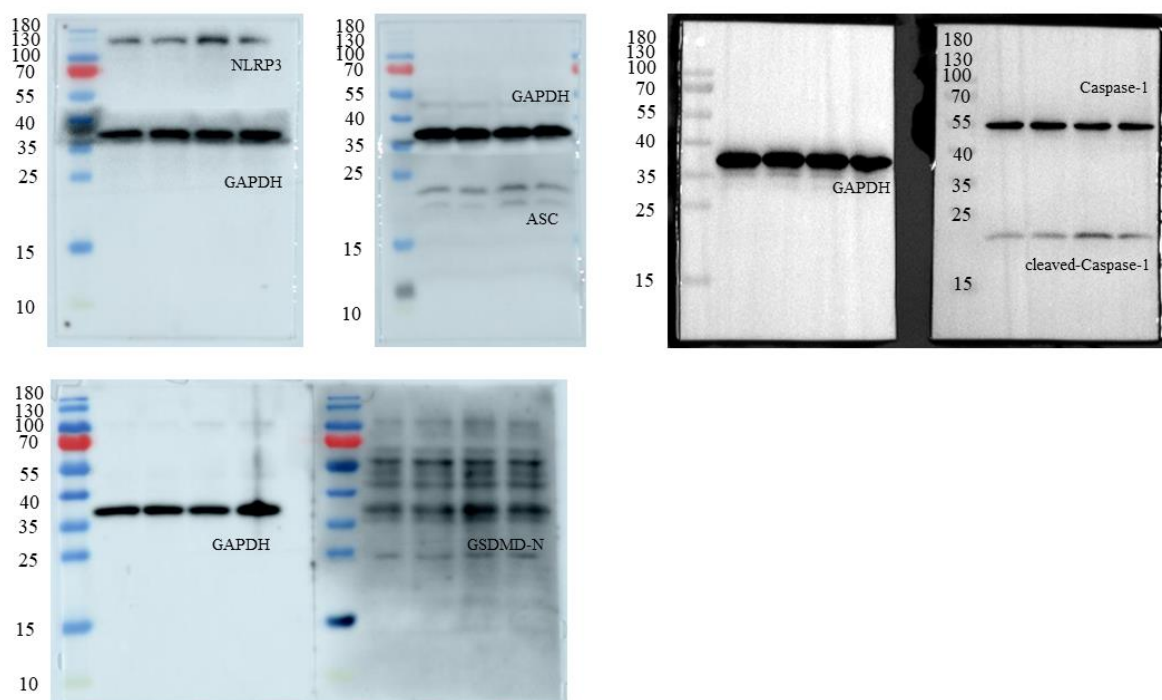

Supplement: Supplementary file 1 [file biomolecules-14-01135-s001.zip › biomolecules-3162187-original-images.pdf]
